# Digital Quantification of Occlusal Contacts: A Methodological Study

**DOI:** 10.3390/ijerph18105297

**Published:** 2021-05-16

**Authors:** Jacob Sigvardsson, Samuel Nilsson, Maria Ransjö, Anna Westerlund

**Affiliations:** Department of Orthodontics, Sahlgrenska Academy, University of Gothenburg, SE-40530 Gothenburg, Sweden; jacob.sigvardsson@vgregion.se (J.S.); samuel.f.nilsson@regionkalmar.se (S.N.); maria.ransjo@odontologi.gu.se (M.R.)

**Keywords:** occlusion, occlusal contacts, digital casts, digital models, orthodontics

## Abstract

Objective: To compare the reliability of digital occlusal contacts quantification and the validity of digital occlusal contacts quantification with traditional methods used for occlusal contact determination. Subjects and Methods: Thirty participants, all of whom were students at the Sahlgrenska Academy, University of Gothenburg in Gothenburg, Sweden, were included in the study. Three different methods were used to evaluate occlusal contacts: (I) a digital examination of the patients’ casts, using the Ortho 3D Models (O3DM) software and measuring the total occlusal contact area in square millimeters (DE); (II) an examination involving the measurement of the total number of occlusal contacts on stone casts mounted in an articulator (AE); and (III) a clinical examination with the measurement of the total number of occlusal contacts with 8 μm-thick articulating foil (CE). Results: The repeated digital measurements (same casts scanned multiple times) showed a significant correlation of 0.85 (*p* < 0.01), which shows a diagnostic consistency. Furthermore, there was a significant correlation between the results obtained with the DE method and the AE of 0.41 (*p* < 0.05), and between those acquired with the AE method and the CE of 0.37 (*p* < 0.05). However, no significant correlation was found between the DE method and the CE method with a correlation coefficient of 0.10 (*p* > 0.05). Conclusions: Digital casts can be used for quantification of the total occlusal contact area (in mm^2^) owing to the high reliability of repeated measurements and the strong validity of the method compared to traditionally employed stone cast measurements.

## 1. Introduction

In layman’s language, an “occlusion” describes the “contact relationships between the teeth of the maxilla and the mandibula in intercuspidation” [1] and the “static relationship between teeth in contact” [2]. In the scientific literature, occlusion has been defined in several ways over the years by various authors. In 1899, Angle described the “key to occlusion” as the relative position of the first molars [3]. In 1969, Ricketts described his view of normal occlusion [4], and in 1976, Andrews described six keys to an ideal static occlusion [5].

The dental occlusion exerts important psychosocial and biological roles [6]. However, there is no definitive evidence in the scientific literature regarding correlations between occlusion and general health, mastication, temporomandibular joint dysfunction (TMD), speech, stability of orthodontic treatment, etc. [7].

The ideal number of occlusal tooth contacts differs widely in the literature, ranging from 11 to 64 [8,9,10,11]. The vast majority of individuals do not have an ideal occlusion. They have a so-called “physiological occlusion”, which does not require any treatment for correction [12]. Dental occlusion is affected by dentist-applied treatments, ranging from fillings to extensive reconstructions and orthodontic treatments [13]. Research has shown that changes in occlusion lead to a neuroplastic modification that is important for adaptation [14]. Thus, in response to peripheral stimuli, the brain has the ability to change, and understanding this phenomenon can enhance our knowledge of patients’ abilities to adapt to new occlusal relationships [15].

Currently, several methods are used to evaluate occlusal contacts. Indicators for evaluating occlusal contacts can be analog or digital in nature, and consist of different types of materials, such as paper and silicon. All of the occlusal indicators vary significantly in terms of their handling and marking characteristics [16,17,18]. In recent years, digital imaging of stone casts has emerged as an alternative to the conventional method [19]. The replacement of stone casts with digital casts has several advantages, such as no need for a non-digital storage area at the clinic, instant accessibility, durability, transferability, and objective measurements [20,21].

Studies that have evaluated the reliability and validity of digital casts have generally reached the same conclusions: that digital models are clinically acceptable, and that the reproducibility is good compared with the traditional stone cast model analysis [22,23,24,25,26,27]. A review article has suggested that the use of digital models as an alternative to traditional measurements performed on stone casts is to be recommended, even though the strength of the evidence presented in the relevant studies varies in quality [21]. It has been reported that computerized occlusal analysis is superior or similar in efficacy to the non-digital methods when it comes to accurately indicating occlusal contacts [28,29]. It also provides an objective analysis for determining occlusions [30].

Study models are essential in orthodontic diagnosis, treatment planning, and evaluations of treatment. Due to the digital format, dentists are able to communicate with colleagues during diagnosis or treatment planning. It is of great importance that the digital models used represent the traditional casts and the intra-oral conditions. Therefore, it is important to evaluate new digital methods to ensure that they are reliable and valid. To the best of our knowledge, no study conducted to date has evaluated the reliability of a digital examination (i.e., measuring the total occlusal contact area in square millimeters) and the validity of recording occlusal contacts, as compared to occlusal contacts observed in vivo and on stone casts mounted in an articulator. The aim of this study was to assess the reliability of the digital quantification of occlusal contacts and evaluate the validity of such quantification in comparison to traditional methods used for occlusal contact determination.

## 2. Subjects and Methods

### 2.1. Sample Description

In total, 30 participants, 13 males and 17 females, with a mean age of 25.3 years (24.5 and 25.9 years, respectively) were included in the study. All the participants were students at the Sahlgrenska Academy, University of Gothenburg, Sweden. All the included participants volunteered for the study.

All the participants had almost normal occlusion with no restorations, with one exception. Due to single contacts on the third molars and an almost full open bite resulting in instable study casts, one participant had to be excluded from the study. All the other participants had no or negligible bite discrepancies.

The participants were informed and given instructions about the procedures included in the study, and written consent was obtained before any measurements were made. This study was approved by the Regional Ethical Committee of Gothenburg (Dnr. 290-09).

The study comprised three measurement stages, with each participant contributing at every stage:

- a clinical examination with counting of the total number of occlusal contacts (CE method);
- a practical study cast examination with the measurement of the total number of occlusal contacts on stone casts mounted in an articulator (AE method);
- a digital examination with the measurement of the total occlusal contact area in millimeters (DE method).

The measurements presented in the results comprise the total number of occlusal contacts from the CE and AE methods, and the total occlusal contact area (in mm^2^) from the DE method.

### 2.2. Production and Scanning of Stone Casts

Alginate impressions of both arches and indexes in the intercuspal position using wax (Alminax wax or Modellvax Tenax) were taken from all participants and were sent to a dental laboratory for the construction of stone casts.

The stone casts were examined for the presence of artefacts on the occlusal surfaces. When artefacts were found they were removed gently.

The stone casts were sent for digital scanning (Ortholab Sp.z o.o., Częstochowa, Poland) to be converted into 3D digital cast models. The digital models were analyzed using the DDP-Ortho ver.1.4 software in O3DM (OrthoLab, Częstochowa, Poland).

### 2.3. Clinical Examination (CE)

In the first stage of the study, all the participants visited the students’ training clinic at the Public Dental Service, University Clinics of Odontology, Gothenburg, to undergo a clinical examination during which their total number of occlusal contacts was registered.

A protocol (see Appendix A) was devised beforehand with all the steps of the clinical examination to ensure that the same protocol was followed for all the participants.

A visual inspection was performed to make sure that all the occlusal surfaces were clean. The participants were then asked to close their mouth repeatedly in the intercuspal position, to make sure that the position was reproducible. Before the start of the examination, a dry area was prepared using parotis rolls. Articulating foil (Troll Foil 8 μm; Trollhätteplast AB, Trollhättan, Sweden) was placed intra-orally in five pre-determined locations, with the participants tapping twice in the intercuspal position at every location (molars bilaterally, premolars bilaterally, and at the front).

**Figure 1:**
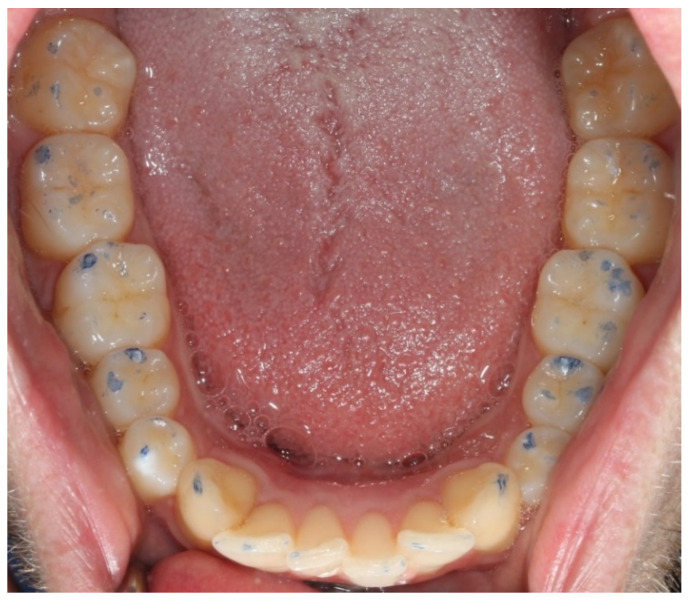
Lower jaw in the clinical examination.

Photographs were taken of the occlusal surfaces of the maxilla and the mandible, respectively, for subsequent evaluation of intra-examiner and inter-examiner reliabilities (Figure 1).

The number of occlusal contacts on each tooth was registered for both jaws. Third molars were not registered (only 4 of the 29 participants had third molars). The number of actual occlusal contacts on each tooth was registered by authors JS and SN, who performed one registration each.

### 2.4. Study Cast Examination (AE)

The models were inserted into an articulator. The articulating foil was placed in the same order and using the same procedure as in the CE method. The number of actual occlusal contacts on each tooth was registered by authors JS and SN, who performed one registration each.

**Figure 2:**
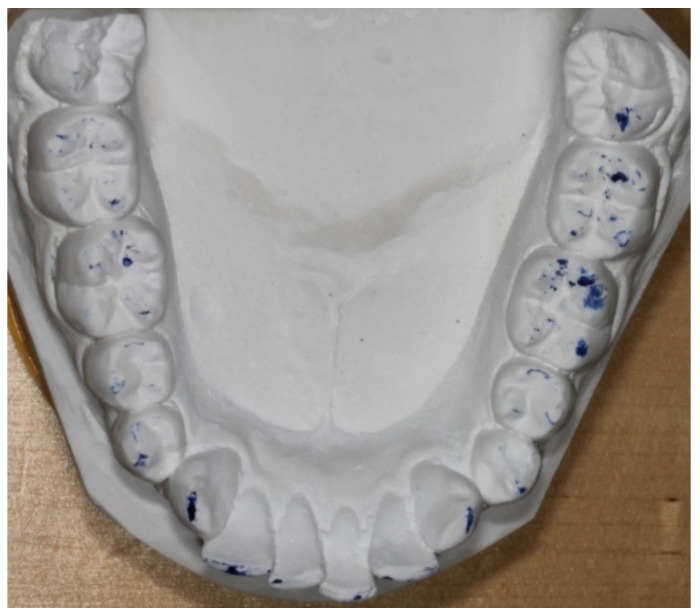
Lower jaw in the study cast examination.

Photographs of the occlusal surfaces of the stone casts were taken for later analysis and for intra-examiner and inter-examiner reliabilities (Figure 2).

### 2.5. Digital Examination (DE)

**Figure 3:**
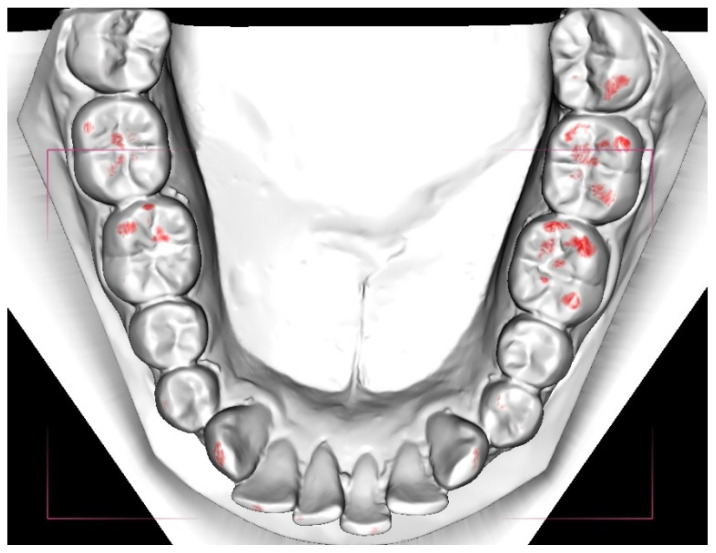
Lower jaw in the digital examination.

The 3D digital cast models were analyzed in the O3DM program DDP-Ortho ver. 1.4. The total area of occlusal contacts was calculated automatically and is presented in units of mm^2^. The cut-off value was set at the lowest value, which was 0–0.2 mm (Figure 3).

Each cast was scanned four times, which resulted in four digital values for each cast. The mean of the four digital values for each scan was used in the comparisons of the methods.

### 2.6. Statistical Analyses

All statistical analyses were performed using the SPSS ver. 23 (SPSS, Chicago, IL, USA) and Microsoft Excel 2010 (Microsoft Inc., Redmond, WA, USA) software packages.

The reliability of the DE method and the validities of the different methods, i.e., correlation, were calculated with Pearson’s correlation (2-tailed) and were presented visually in the form of scatter plots.

### 2.7. Error of Method

The reproducibility levels of the measurements (intra-examiner and inter-examiner) were tested with the Dahlberg’s formula and the intra-class correlation (ICC), respectively.

In order to study the reliability of the clinical examination, participants were randomly selected to undergo a second clinical examination after 2 weeks.

## 3. Results

**Figure 4:**
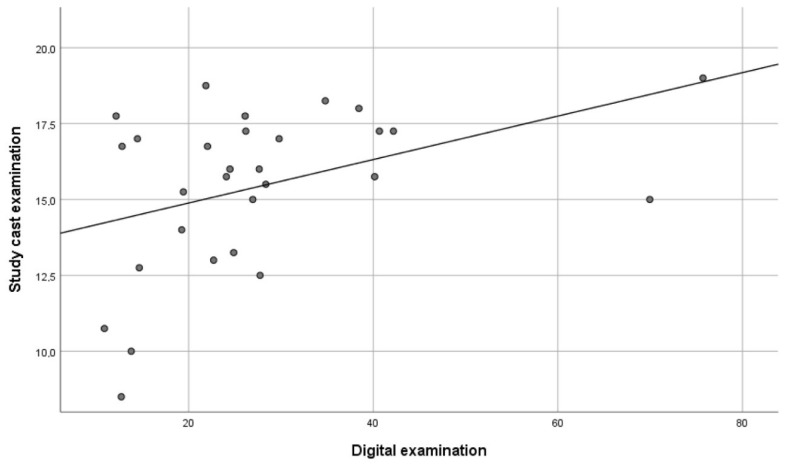
Scatterplot explaining the relationship between the total amount of occlusal contacts in the study cast examination and the total occlusal contact area in mm^2^ in the digital examination (*n* = 29). Every dot represents one of the included participants (*p* < 0.05).

**Figure 5:**
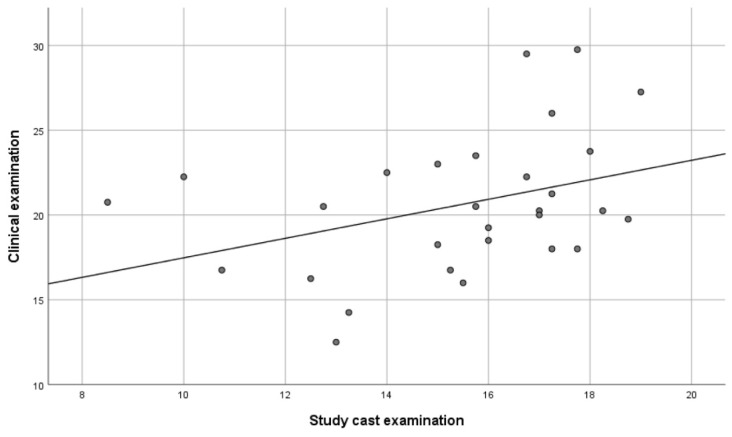
Scatterplot explaining the relationship between the total amount of occlusal contacts in the clinical examination and the total amount of occlusal contacts in the study cast examination (*n* = 29). Every dot represents one of the included participants (*p* < 0.05).

**Figure 6:**
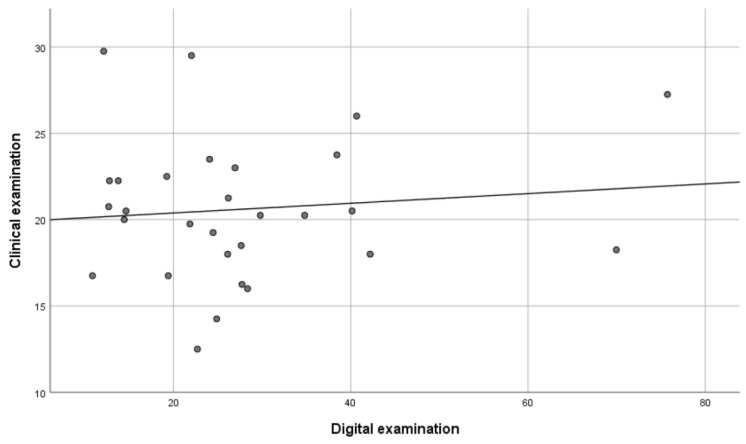
Scatterplot explaining the relationship between the total amount of occlusal contacts in the clinical examination and the total occlusal contact area in mm^2^ in the digital examination (*n* = 29). Every dot represents one of the included participants.

The repeated digital measurements showed a significant correlation of 0.85 (*p* < 0.01), indicating that the reliability of the method was high. Furthermore, there was a significant correlation between the DE method and the AE method of 0.41 (*p* < 0.05) (Figure 4), and between the AE method and the CE method of 0.37 (*p* < 0.05) (Figure 5). However, no significant correlation (0.10) was found between the DE method and the CE method (Figure 6).

The relationships between the different measurements are shown in scatterplots where every dot represents one of the participants. The x-axis and the y-axis each represent one of the examination methods with its measured value (the total amount of occlusal contacts or the total occlusal contact area in mm^2^).

The intra-examiner and inter-examiner reliability levels showed a good to very good conformity within and between the examiners regarding all the measurements made in the study (Table 1). The repeated clinical examination revealed good intra-examiner reliability (0.75).

When using the different methods, the number of occlusal contacts was highest in the molar region, followed by the premolar and front region for almost all participants. Moreover, the number of occlusal contacts in the molar region was observed to be higher with the CE method compared to the AE method. However, there was no major difference in the premolar or front region (canine to canine) between the CE and AE methods. The higher amount of total contacts using the CE method resulted in a higher risk of deviation in the total amount of occlusal contacts compared to the AE method.

## 4. Discussion

This study demonstrated that digital models for the quantification of occlusal contacts represent a reliable and valid method for measuring occlusal contacts. The study was comprised of 30 participants whose occlusal contacts were evaluated with three different methods: (I) a digital examination using the Ortho 3D Models (O3DM) software and measuring the total occlusal contact area in square millimeters (DE); (II) an examination involving the measurement of the total number of occlusal contacts on stone casts mounted in an articulator (AE); and (III) a clinical examination with the measurement of the total number of occlusal contacts (CE). The correlation between the different methods was calculated with Pearson’s correlation (2-tailed) and was presented visually in the form of scatter plots.

Although there have been many reports on the reliability and validity of different digital programs, few have considered occlusal contacts as a variable, and no study has used clinical registrations as a control. Most of the studies conducted to date have evaluated occlusion or occlusal contacts in a larger context, e.g., in the ABO index. Nonetheless, some earlier studies have reported that digital evaluation of occlusal contacts works well [19,22,23,24,27]. O3DM has, to the best of our knowledge, never been evaluated with respect to occlusal contacts or occlusion.

The digital software program used for the quantification of occlusal contacts demonstrated good reliability with repeated measurements. Regarding validity, there were significant correlations between the CE and AE, and between the AE and DE measurements. However, there was no correlation between DE and the CE measurements.

The conclusion that the program is valid is based on the observed correlation between the DE and AE measurements. All the studies to date that have evaluated the validity of digital measurements for different variables have carried out measurements on study casts as the control and gold standard [21,22,23,24,25,26]. However, there are problems associated with clinical registrations of occlusal contacts. When comparing the number of contacts between the CE and AE methods, there was a decrease in the number of occlusal contacts in 28 out of 29 of the participants. It has been observed that the number of occlusal contacts recorded in the mouth is generally higher than the number recorded in articulators [31]. Studies have reported that variables, such as the thickness of the material and moisture content, all affect the markings in both number and size [16,17,18]. A high degree of false-positive markings, poor reproducibility, and variations in the size and number of the markings are the outcomes of these variables [17,18,32]. The evaluation should also be as independent as possible from subjective interpretation by clinicians [16,32]. The moisture content, for example, is a factor in the clinical examination, which may explain in part why there is a loss of occlusal contacts when comparing the two measurements, i.e., there are more false-positive markings in the clinical examination. Another complication is that the levels of force used in the two different measurements may not have been the same, which might affect the number and size of the occlusal markings. The observed discrepancy might alternatively be explained by the fact that the TMJ is fixed when the stone casts are mounted in the articulator during the study cast examination, whereas the joint is not fixed during the clinical examination.

However, even though the clinical registration was complicated, the repeated measurement showed good agreement.

The intra-examiner and inter-examiner reliability scores have, in similar studies, been reported to range from good to excellent [19,22,23,24,25,26,27]. The ICC score for intra-examiner reliability in this study was considered to be good to very good compared with earlier studies. The ICC score for inter-examiner reliability in this study is considered to be very good, although there are no relevant earlier studies to compare it with.

Stringent requirements must be placed on the stone casts and indexes when the digital models are created by scanning. When fewer occlusal contacts are present, for example, in an open bite situation, the reliability of the measurements decreases. Therefore, the potential misuse of the index used to register the relationship between the jaws represents a possible error. Furthermore, a well-produced stone cast is of the highest importance, and thus also of importance is the impression with alginate. In this study, the stone casts’ heels were ground with the help of the acquired index.

The digital software used to quantify the occlusal contacts within an area is based on a method that divides the contact surface into a number of triangles, which is later converted to an area in mm^2^. When the software compares the mandible and the maxilla, the forms of the triangles differ slightly. As a consequence, when the values in mm^2^ are extracted, the values will differ slightly. The total areas measured in the maxilla and the mandible have, therefore, been summed and then divided by two, as the total area of the occlusal contacts should be approximately the same in both jaws. Therefore, the total number of occlusal contacts also has been divided by two, both in the clinical examination and in the study cast examination (one contact in the maxilla should represent one in the mandible).

It is debatable whether it is more important to know the total number of occlusal contacts or the total area of the occlusion. Using the software, the total area of the occlusion or occlusal contacts measured in mm^2^ can be extracted. This means that two cases might have the same total area of occlusion even though the numbers, and thus even the distribution, of occlusal contacts differ. However, the method is very illustrative in that it shows the area of occlusion.

The new digital instrument will make it easier to apply occlusion or occlusal contacts in orthodontic planning and decision making, as well as in the evaluation of treatment. Reliable and valid modern digital methods to quantitate occlusal contacts will facilitate and increase the possibilities of studying occlusion. It will eventually create a better understanding of the importance of occlusion, especially with respect to the relationships between dental occlusion and general health, TMD, mastication, speech, stability of orthodontics, etc. [7,33].

### Limitation

A way to avoid sources of errors linked to the process of impression to scanning of the stone cast would be to use an intra-oral scanner directly in the mouth of the participant, a technique that is becoming more common in dentistry. This would eliminate potential errors with the impressions, indexes, damage during shipping, etc.

## 5. Conclusions

Digital casts might be used for quantification of occlusal contacts in mm^2^. However, it is of the utmost importance that the study cast and index are of high quality to ensure that they are scanned in the right position.

## Figures and Tables

**Table 1:** Intra-examiner and inter-examiner reliability scores tested with the ICC. The scores show that there is good conformity within and between both examiners.

Intra-examiner		ICC	ICC–CI 95%
**Examiner 1**	- Clinical examination	0.909	0.810–0.956
	- Study cast examination	0.797	0.147–0.929
**Examiner 2**	- Clinical examination	0.939	0.799–0.976
	- Study cast examination	0.862	0.558–0.945
**Inter-examiner**		**ICC**	**ICC–CI 95%**
**Clinical examination**		0.939	0.847–0.973
**Study cast examination**		0.935	0.865–0.969

## Data Availability

The data presented in this study are available on request from the corresponding author.

## Appendix A

**Protocol–Clinical measurement of occlusal contacts**
Materials–parotisroll (for moisture control), disposable air/water syringe tip, articulating paper (Trollfolie, 8 µm), articulating foil forceps, camera, mirrors for intraoral photos, dental retractors, mouth mirrors
  1. Inspection of cleanliness of the participant’s teeth
  2. Verify that the participant is able to repeat the same intercuspid position
  3. Moisture control
    - 2 parotisrolls
    - Verify once more that the participant is able to repeat same intercuspid position (with the parotisrolls in place)
    - Blasting with syringe tip
  4. Occlusal contacts with articulating paper (Trollfolie, 8µm)
    - Articulating foil forceps with articulating foil is applied in the following order:
      - Molars on the left side
      - Premolars on the left side
      - Front teeth
      - Premolars on the right side
      - Molars on the right side
    - 2 bites in intercuspid position in every region
    - Avoid to drag the articulating foil on the teeth’s surfaces
  5. Photos on occlusal contacts
    - Warming up intraoral mirrors with hot water
    - Applying dental retractors
    - Photos of the upper and lower jaw
  6. Clinical measurement of occlusal contacts
    - JS & SN counts the occlusal contacts separately and the number of contacts is inserted in to a registration form
    - The number of occlusal contacts on each tooth is recorded
    - The order of counting contacts is quadrant 1 →2 → 3 → 4
